# An Analysis of the Precision and Reliability of the Leap Motion Sensor and Its Suitability for Static and Dynamic Tracking

**DOI:** 10.3390/s140203702

**Published:** 2014-02-21

**Authors:** Jože Guna, Grega Jakus, Matevž Pogačnik, Sašo Tomažič, Jaka Sodnik

**Affiliations:** Faculty of Electrical Engineering, University of Ljubljana, Tržaška 25, Ljubljana 1000, Slovenia; E-Mails: grega.jakus@fe.uni-lj.si (G.J.); matevz.pogacnik@fe.uni-lj.si (M.P.); saso.tomazic@fe.uni-lj.si (S.T.); jaka.sodnik@fe.uni-lj.si (J.S.)

**Keywords:** Leap Motion Controller, motion capture system, precision measurement, spatial distortion measurement

## Abstract

We present the results of an evaluation of the performance of the Leap Motion Controller with the aid of a professional, high-precision, fast motion tracking system. A set of static and dynamic measurements was performed with different numbers of tracking objects and configurations. For the static measurements, a plastic arm model simulating a human arm was used. A set of 37 reference locations was selected to cover the controller's sensory space. For the dynamic measurements, a special V-shaped tool, consisting of two tracking objects maintaining a constant distance between them, was created to simulate two human fingers. In the static scenario, the standard deviation was less than 0.5 mm. The linear correlation revealed a significant increase in the standard deviation when moving away from the controller. The results of the dynamic scenario revealed the inconsistent performance of the controller, with a significant drop in accuracy for samples taken more than 250 mm above the controller's surface. The Leap Motion Controller undoubtedly represents a revolutionary input device for gesture-based human-computer interaction; however, due to its rather limited sensory space and inconsistent sampling frequency, in its current configuration it cannot currently be used as a professional tracking system.

## 1. Introduction

The user interface and the corresponding interaction modalities play an essential role in the human-computer relationship. Advanced multimodal interfaces present yet another step in this equation, providing users with the freedom and flexibility to choose the best input modality for specific tasks. Users generally prefer multimodal interaction when it is available and intuitive to use [1,2].

Gesture-based user interfaces, in combination with the latest technical advances that incorporate accurate yet affordable new types of input devices, provide realistic new opportunities for specific application areas (e.g., entertainment, learning, health, engineering), especially for users who are uncomfortable with more commonly used input devices and/or technology [3].

Gesture input devices and sensors are of special interest. Gesture acquisition methods can, in general, be divided into methods incorporating a specific device that the user must physically hold or have on his/her body and hands/body-free methods. The latter become more and more popular as the user becomes a controller rather than an operator. One of the first widespread, accurate, and commercially viable solutions was the Nintendo WiiMote controller, bundled with the Wii console, released in 2006. The WiiMote, besides its vocal and haptic modalities, incorporates an accelerometer that allows the acquisition of full 3D gestures. It can operate as a separate device and has been successfully used for many atypical applications [4]. Another important milestone is the Microsoft Kinect sensor, an add-on for the Xbox 360 console, which was released in late 2010. The Kinect, among its visual and auditory inputs, includes a depth-sensing camera. In combination with an open SDK, it can be used to acquire and recognize full body gestures for multiple users at a time [5]. The latest technological breakthrough in gesture-sensing devices has come in the form of a Leap Motion Controller (Leap Motion, San Francisco, CA, United States) [6]. The controller, approximately the size of a box of matches, allows for the precise and fluid tracking of multiple hands, fingers, and small objects in free space with sub-millimeter accuracy. According to [7], the Leap Motion Controller represents a major leap in input technology that could, with its enhanced interaction possibilities, trigger a new generation of far more useful 3D displays and possibly surpass the mouse as a primary input device.

The main goal of the research presented in this paper was to analyze the precision and reliability of the Leap Motion Controller in static and dynamic conditions and to determine its suitability as an economically attractive finger/hand and object tracking sensor. The evaluation was performed with the aid of a high-speed and highly accurate optical motion capture system. To the best of the authors' knowledge, no study has yet been conducted with the Leap Motion Controller in combination with an optical motion capture system.

The main contributions of this paper are analyses of the following:

- Precision and reliability (spatial dispersion of measurements through time) of the controller
- The spatial distortion of accuracy (variation of accuracy in different regions of sensory space)
- Sampling frequency and its consistency.

The rest of the paper is organized as follows: following the Introduction, previous related work is presented in Section 2. The experimental environment, measurement methodology, and measurement scenarios used in this study are described in Section 3. The detailed results are analyzed and discussed in Section 4. Finally, key conclusions are drawn and recommendations are offered in Secion 5.

## 2. Related Work

To illuminate the choice of motion capture system in combination with the Leap Motion Controller, the results of the research in [8] are discussed. The authors of that study focused on the adaptive gesture recognition system while developing a gesture database to eliminate the individual factors that affect the efficiency of the recognition system. In particular, hand gestures were investigated. To acquire input data for the experiment, a Qualisys™ Motion Capture System [9] was used, similar to the one in our own setup.

The Microsoft Kinect sensor was developed with hand/arm and full-body gesture recognition in mind. The authors of [10] provide a detailed analysis of the accuracy and resolution of the Kinect sensor's depth data for indoor mapping applications. The experimental results show that the random error in depth measurement increases with increasing distance to the sensor, ranging from a few millimeters to approximately 4 cm at the maximum range of the sensor. The quality of the data is also found to be influenced by the low resolution of the depth measurements. The obtained accuracy is, in general, sufficient for detecting arm and full body gestures, but is not sufficient for precise finger gestures such as handwriting. The input device latency and spatial jitter are also important factors [11].

The Leap Motion Controller presents a milestone in consumer finger/object and gesture tracking input technology. The device itself was made publicly available in summer 2013, and therefore not much scientific work has been published yet. In [12], the authors describe an application of the Leap Motion Controller for the direct manipulation of an industrial robot arm with six axes of freedom. The Leap Motion Controller is used for finger position tracking. To increase the tracking precision, an interpolation of the acquired data is performed using polynomial splines. The aim of the research was to reproduce complex tasks in 3D without constraints on the operator. This goal reflects the importance of gesture-based interfaces that utilize low-cost, consumer-grade input sensor devices for industrial use.

Another study of the Leap Motion Controller in [13] shows its potential in gesture and handwriting recognition applications. The acquired input data are treated as a time series of 3D positions and processed using the Dynamic Time Warping algorithm. The authors report promising recognition accuracy and performance results.

In [14], a novel interface approach that combines 2D video-based augmented reality with a partial voxel model to allow more convincing interactions with 3D objects and worlds is presented. The interface enables users to interact with a virtual environment through a hand-controlled interface and allows for correct mutual occlusions between the interacting fingers and the virtual environment. A combination of the Leap Motion Controller and a webcam is used to track the users' fingers and overlay the appropriate video for an augmented view.

Finally, in [15], the authors present a study of the accuracy and robustness of the Leap Motion Controller. An industrial robot with a reference pen allowing suitable position accuracy was used for the experiment. The results show a deviation between the desired 3D position and the average measured positions below 0.2 mm for static setups and of 1.2 mm for dynamic setups.

## 3. Experimental Design

The controller's performance was evaluated through two types of measurements. In the first measurement, a series of fixed static points in space were tracked and recorded for a longer period of time to evaluate the consistency and dispersion of the results. The coordinates of the points were systematically chosen to cover the majority of the controller's sensory space. In the second measurement, a constant distance was provided between two objects, which were then moved freely around the sensory space. The tracking accuracy of the controller was then evaluated based on the distortion of the distance between the two objects. The reference system (a professional optical motion capture system) was used to determine the exact spatial positions of the tracked objects and the distances between them.

### 3.1. The Leap Motion Controller

**Figure 1.**
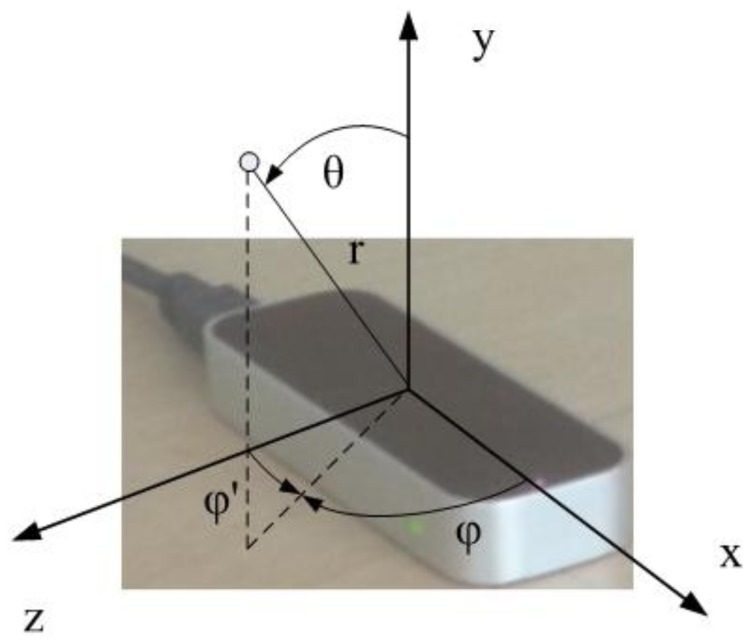
The Cartesian and spherical coordinate systems used to describe positions in the controller's sensory space.

The Leap Motion Controller uses infrared (IR) imaging to determine the position of predefined objects in a limited space in real time. Technically, very few details are known about the precise nature of the algorithms used due to patent and trade secret restrictions. However, from inspection of the controller, it is clear that three separate IR LED emitters are used in conjunction with two IR cameras. Therefore, the controller can be categorized as an optical tracking system based on the stereo vision principle. According to the official information [6], the Leap software analyzes the objects observed in the device's field of view. It recognizes hands, fingers, and tools, reporting discrete positions, gestures, and motion. The controller's field of view is an inverted pyramid centered on the device. The effective range of the controller extends from approximately 25 to 600 millimeters above the device (1 inch to 2 feet). The controller itself is accessed and programmed through Application Programming Interfaces (APIs), with support for a variety of programming languages, ranging from C++ to Python. The positions of the recognized objects are acquired through these APIs. The Cartesian and spherical coordinate systems used to describe positions in the controller's sensory space are shown in Figure 1. However, it should be noted that the sampling frequency is not stable, cannot be set, and varies significantly.

### 3.2. The Reference System

A high-precision optical tracking system [9] consisting of eight Oqus 3+ high-speed cameras and Qualisys Track Manager software (version 2.8—build 1065) was used as the reference system (Qualisys Inc., Gothenburg, Sweden). Such systems are widely used for the fast and precise tracking of various objects in industrial applications, biomechanics, and media and entertainment applications. The tracking precision depends on the number of cameras used, their spatial layout, the calibration process, and the lighting conditions. In our case, only three markers were used, one for static measurement and two for dynamic measurement. In the dynamic measurement, a simple Automatic Identification of Markers (AIM) model was created from the two selected markers and their connecting bone. All markers were seen by all cameras at all times. The standard deviation of the noise for the static marker was measured for each individual coordinate: std*_x_* = 0.018 mm, std*_y_* = 0.016 mm and std*_z_* = 0.029 mm.

### 3.3. Technical Setup

The Leap Motion controller was placed on a table 60 × 60 cm in area and 73 cm in height. The controller was firmly attached to the table, ensuring no undesired movement of the device. The controller transmitted data on the identified objects to a desktop computer (Intel^®^ Core™ i7-2600 CPU 3.40 GHz with 8 GB of RAM). A set of scripts was written in the Python programming language using the Leap Motion APIs specifically for this study. The scripts were used for real-time data acquisition and logging. The operation of the controller was monitored in real time using the Leap Motion Visualizer software.

The optical reference system provided a calibrated measurement volume of approximately 1 × 1 × 1 m in size, with a resolution of 1.3 million pixels and a constant frame rate of 500 frames per second. The cameras were set up uniformly, encircling the Leap Motion controller so that each camera's point of view was directed towards the controller. A set of hard passive markers with diameters of 12.5 mm was used in the measurements. The coordinate systems of the reference system and the controller were aligned at the origin of the controller's coordinate system.

Two types of measurements were performed within the experiment, under two experimental conditions:

- Static conditions: acquisition of a limited number of static points in space
- Dynamic conditions: tracking of moving objects with constant inter-object distance within the calibrated space

**Figure 2.**
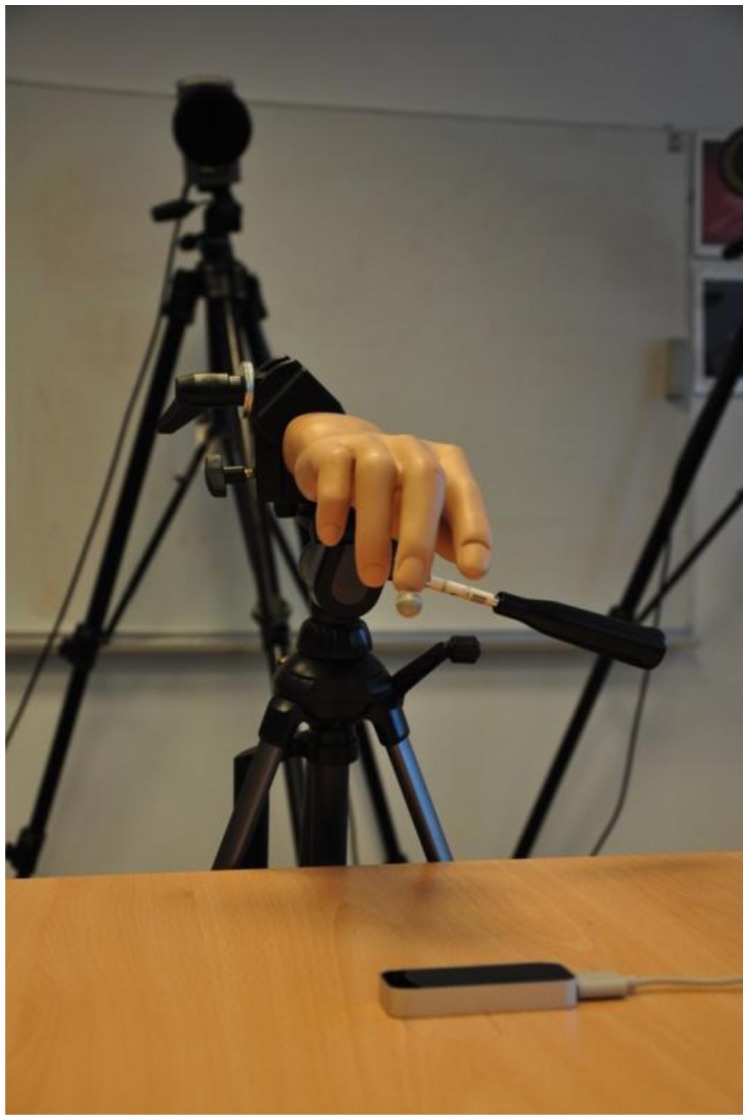
The setup of the experimental environment.

Our pre-experiment trials indicated the controller's inability to track static objects that do not resemble the human hand. We can only speculate that this limitation is due to the controller's internal algorithms, as they are protected by patents and therefore not publicly disclosed. A pointed object, such as a pen tip (used for tracking in [15]), was successfully tracked only if constantly in motion. When it was stationary and mounted on a stand, it was successfully tracked for only approximately 8–10 s. After this period of time, the measurement was automatically stopped by the controller. Therefore, a plastic arm model was used (Figure 2) instead of a simpler object.

**Figure 3.**
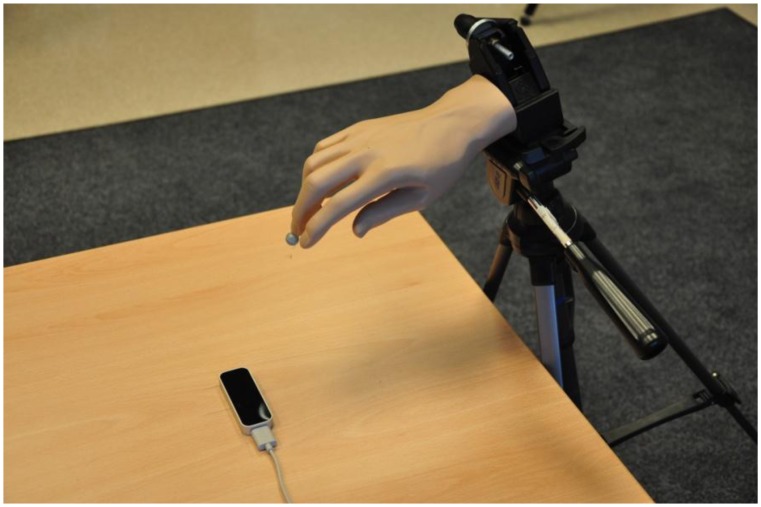
To improve the tracking capabilities of the Leap Motion Controller, the marker was placed at the tip of the index finger of a plastic arm model. During the measurement of static locations, the arm was fixed in place using a stand.

During the measurement of static locations, the arm model was firmly attached using a stand (Figure 3) and directed perpendicular to the *z* = 0 plane in the opposite direction from the *z* axis. Additionally, a reflective marker was attached to the index fingertip of the plastic arm for simultaneous tracking by the controller and by the reference motion capture system. The stability of the stand was measured using the reference system, which indicated the dispersion of the measured index fingertip location to be below 20 μm.

**Figure 4.**
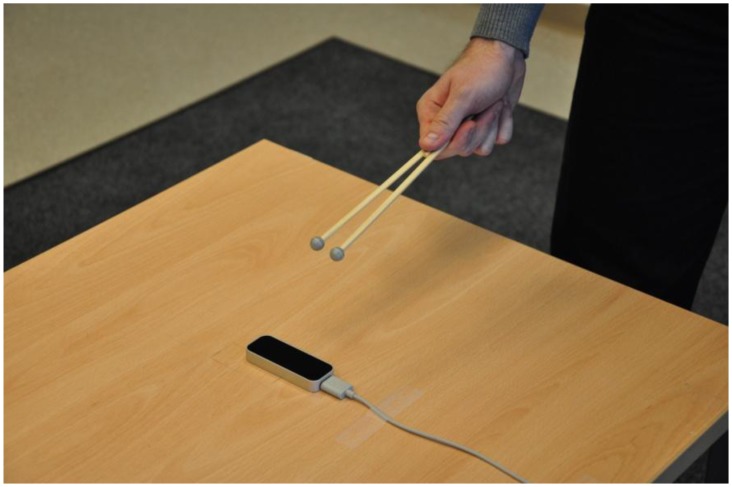
The V-tool used for dynamic measurements.

For dynamic measurements, the tracking objects were moved around the sensory space with an approximately constant speed of 100 mm/s. Instead of the plastic arm, a special tool was used to mimic two human fingers. It consisted of two wooden sticks with markers fixed together to form a V-shape (hereafter: “the V-tool”) (Figure 4). This tool provided a constant distance between the two tracked objects, which was used to evaluate the tracking performance. It was perfectly tracked by the controller and the reference system simultaneously. The exact distance was acquired using the reference system (*d* = 21.36 mm, std*_d_* = 0.023 mm). The arm model with five fingers proved to be very impractical for this type of measurement, as the controller usually tracked the five fingers as five individual points that could not be identified separately. It was therefore almost impossible to identify the results for two selected fingers and calculate their inter-distance.

### 3.4. The Methodology

All measurements were conducted in an environment with a constant temperature of 22 °C and an illumination intensity of approximately 500 lux, a common legal requirement for the workspace. The sampling frequency of the reference system was set to 500 Hz.

#### 3.4.1. Static Measurements

**Figure 5.**
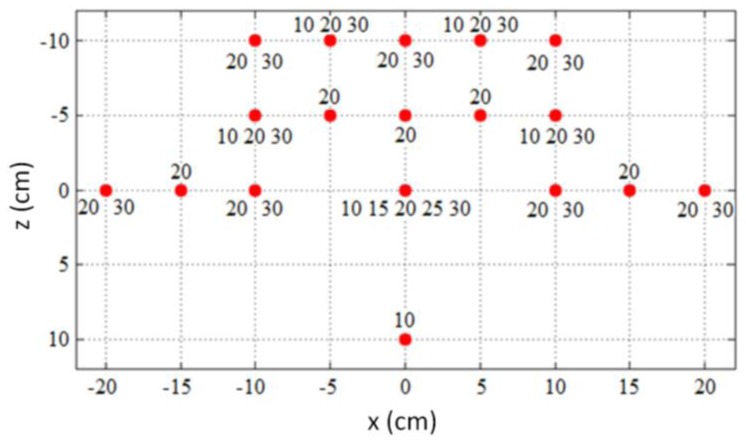
The measurement grid displaying the reference locations of the static measurements.

The 37 reference locations where static measurements were performed are shown in Figure 5. The locations of the reference points were selected systematically to cover the majority of the sensory space of the controller. The number at each location in the figure indicates the height (the position on the *y* axis in cm) at which the individual measurements were taken. The actual measurements were taken close to the reference point in the measurement grid with an offset less than 5 mm. At least 4,000 samples were measured at each location. A total of 214,546 samples were obtained for the entire sensory space.

It was initially planned to take measurements along a 3-dimensional grid with 5 cm spacing between the measured locations. However, the pre-experiment trials revealed that it is difficult to obtain stable tracking of static objects at some locations, especially locations in front of the controller (*z* > 0). The measurement grid was therefore modified to include only locations at which the controller was able to provide stable tracking over a longer time period.

The analysis of the collected samples was primarily focused on evaluating the dispersion—a temporal distribution—of the recorded locations for each reference location, which characterizes the repeatability of measurements at a particular location in the controller's sensory space. Repeatability characterizes the ability to relocate the same location in a series of sequential measurements.

For the purpose of the analysis and the presentation of its results, the following mathematical operations and notions are used.

The measured positions are denoted by a set *p*[*i,j*]=(*p_x_*[*i,j*], *p_y_*[*i,j*], *p_z_*[*i,j*]) ∈ *R*^3^, where the components *p_x_*[*i,j*], *p_y_*[*i,j*] and *p_z_*[*i,j*] represent the coordinates in the Cartesian coordinate system of the *j*-th sample (1 ≤ *j* ≤ *N_i_*, *j* ∈ *N*) taken at the *i*-th position (1 ≤ *i* 37,*i* ∈ *N*), and *N_i_* stands for the total number of samples taken at the *i*-th position.

The standard deviation of the *i*-th three-dimensional spatial position is calculated by:

(1) $$\text{std}[i]=\sqrt{\frac{1}{N_{i}-1}\sum_{j=1}^{N_{i}}{\left(E[i,j]-\bar{E[\iota ]}\right)}^{2},}\text{where}$$

(2) $$E[i,j]=\sqrt{{\left(p_{x}[i,j]-\bar{p_{x}[\iota ]}\right)}^{2}+{\left(p_{y}[i,j]-\bar{p_{y}[\iota ]}\right)}^{2}+{\left(p_{z}[i,j]-\bar{p_{z}[\iota ]}\right)}^{2}}$$

where $\bar{p_{x}[\iota ]}$, $\bar{p_{y}[\iota ]}$ and $\bar{p_{z}[\iota ]}$ represent the average coordinates calculated by the arithmetic mean over *N_i_* samples.

$\bar{E[\iota ]}$ stands for the arithmetic mean of *E*[*i,j*] over *N_i_* samples. The standard deviations for individual coordinates (*std_x_*[*i*], *std_y_*[*i*], and *std_z_*[*i*]) were calculated by:

(3) $${\text{std}}_{x}[i]=\sqrt{\frac{1}{N_{i}-1}\sum_{j=1}^{N_{i}}{\left(p_{x}[i,j]-\bar{p_{x}[\iota ]}\right)}^{2}}$$

(4) $${\text{std}}_{y}[i]=\sqrt{\frac{1}{N_{i}-1}\sum_{j=1}^{N_{i}}{\left(p_{y}[i,j]-\bar{p_{y}[\iota ]}\right)}^{2}}$$

(5) $${\text{std}}_{z}[i]=\sqrt{\frac{1}{N_{i}-1}\sum_{j=1}^{N_{i}}{\left(p_{z}[i,j]-\bar{p_{z}[\iota ]}\right)}^{2}}$$

For the analysis in a spherical coordinate system, the following three conversion equations were used (note that, according to the controller's coordinate system, the *y* and *z* axes are mutually switched compared to the standard Cartesian system; therefore, *y* represents the height, and *z* represents the depth):

(6) $$r=\sqrt{x^{2}+y^{2}+z^{2}}$$

(7) $$\theta =\arccos(\frac{y}{r})\text{and}$$

(8) $$\varphi =\{\begin{array}{cc} \arctan(\frac{z}{x}), & x>0\\ \arctan(\frac{z}{x})+\pi , & z\geq 0,x<0\\ \arctan(\frac{z}{x})-\pi , & z<0,x<0\\ \frac{\pi }{2}, & z>0,x=0\\ -\frac{\pi }{2}, & z<0,x=0\\ \text{not defined}, & z=0,x=0 \end{array}$$

Assuming symmetry in the controller's performance over the *x* and *z* axes, we additionally define the modified azimuth angle as follows:

(9) $$\varphi \prime =\{\begin{array}{cc} \arctan\left(\left|\frac{x}{z}\right|\right), & z\neq 0\\ \frac{\pi }{2}, & x\neq 0,z=0\\ \text{not defined}, & x=0,z=0 \end{array}$$

The angle φ′ is measured from the x axis (not from the *z* axis, as in the case of the φ angle) and the line connecting the coordinate origin with the projection of the measured location in the *x*-*z* plane. As the angle φ′ is defined under the assumption of symmetry in the controller's performance over the *x* and *z* axes, it is therefore defined in the range of (0, $\frac{\pi }{2}$) rad.

#### 3.4.2. Dynamic Measurements

In the dynamic measurements, the experimenter moved the V-tool randomly but with a constant speed within the selected region of the controller's sensory space. The V-tool was held in the fist (Figure 4) to simulate two extended fingers and was therefore detected by the controller. The moving speed of the V-tool was approximately 100 mm/s.

The measured sensory space included a volume of 100,000 cm^3^ (−250 mm < *x* < 250 mm, −250 mm < *z* < 250 mm and 0 mm < *y* < 400 mm). This space was systematically covered in a series of four continuous measurements, each covering a layer approximately 100 mm in height (*y* dimension). The data from the individual measurements were then combined for analysis. A total of 119,360 valid positions were recorded with an average density of 1.2 samples per cm^3^.

The primary goal of the dynamic measurements was to evaluate the distortion of the controller's perception of space. As previously explained, the distortion was measured as the deviation of the distance between the two markers located at the tips of the V-tool. The distance between the markers at the *i*-th position was defined as:

(10) $$d[i]=\sqrt{{\left(p_{x1}[i]-p_{x2}[i]\right)}^{2}+{\left(p_{y1}[i]-p_{y2}[i]\right)}^{2}+{\left(p_{z1}[i]-p_{z2}[i]\right)}^{2}}$$

where *p_x_*_1_[*i*],*p_y_*_1_[*i*] and *p_z_*_1_[*i*] represent the coordinates of the first marker, and *p_x_*_2_[*i*],*p_y_*_2_[*i*] and *p_z_*_2_[*i*] represent the coordinates of the second marker. The exact location of the *i*-th position was defined as a central point on the line between the two markers, obtained from the reference tracking system:

(11) $$p_{x\_\text{ref}}[i]=\frac{\left(p_{x1\_\text{ref}}[i]+p_{x2\_\text{ref}}[i]\right)}{2}$$

(12) $$p_{y\_\text{ref}}[i]=\frac{\left(p_{y1\_\text{ref}}[i]+p_{y2\_\text{ref}}[i]\right)}{2}$$

(13) $$p_{z\_\text{ref}}[i]=\frac{\left(p_{z1\_\text{ref}}[i]+p_{z2\_\text{ref}}[i]\right)}{2}$$

## 4. Results

This section presents the measurement results based on the experimental design described in the previous section. The results of the static measurements are presented first, followed by the results of the dynamic measurement scenario.

### 4.1. Static Measurements

The upper two rows in Table 1 show the minimum and maximum standard deviations of the measured static positions. The standard deviations are given for the individual axes as well as for the three-dimensional spatial position. The lower two rows show the spatial positions with the minimal and maximal standard deviations for the individual axes.

The lowest standard deviation (0.0081 mm) was measured on the *x* axis 30 cm above the controller, while the highest standard deviation (0.49 mm) was measured on the *y* axis at the leftmost and topmost positions.

**Figure 6.**
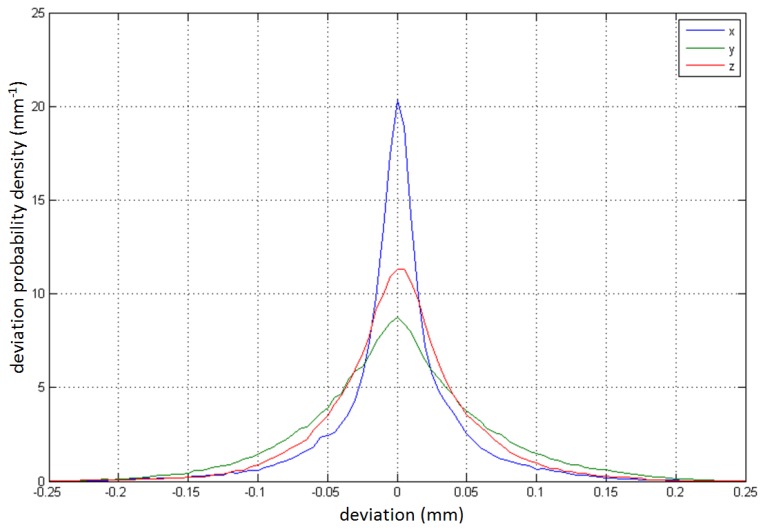
The probability density of the deviations, including all 37 locations.

Figure 6 shows the probability density of the deviation for the individual axes. By deviation, we mean the difference of all the measured samples from their corresponding mean measured positions (*p*[*i,j*]− $\bar{p[\iota ]}$, 1 ≤ *i* ≤ 37, 1 ≤ *j* ≤ *N_i_*). The figure therefore indicates the deviation probability on each of the three axes when making a single measurement in the controller's sensory space. The narrowness and height of the individual curve can therefore be directly interpreted as the consistency of the controller's individual dimensions for tracking static spatial points.

**Figure 7.**
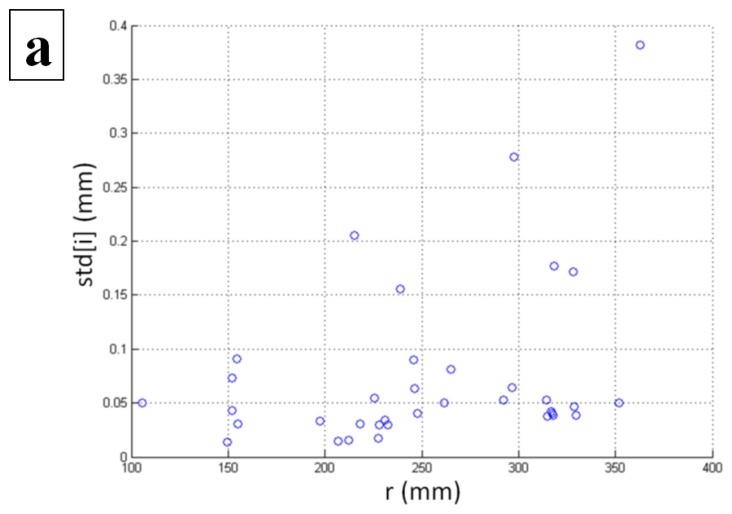

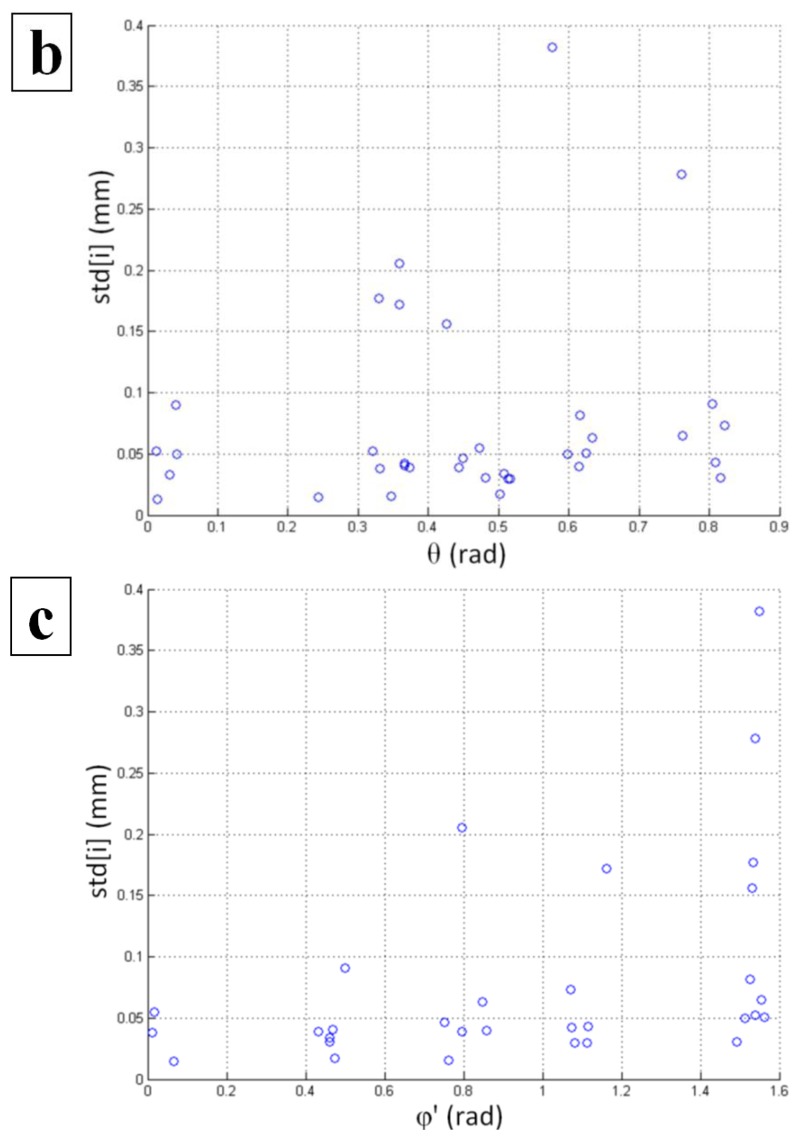
Spatial dependency of (**a**) the radius—*r*; (**b**) inclination—θ; and (**c**) azimuth—φ′ on the standard deviation.

Our further study was focused on the determination of the spatial dependency of the standard deviation of the static measurements. For this purpose, a spherical coordinate system was used instead of the Cartesian coordinate system. The following figures (Figure 7) show the impact of the radius (*r*), inclination (θ), and azimuth (φ′) on the standard deviation. As the angle φ′ is not defined for the *y* axis, the five locations above the coordinate origin with *x* = 0 and *z* = 0 were excluded from the analysis involving the azimuth angle.

The figures indicate a dependency of the standard deviation on the radius and the azimuth. In both cases, the standard deviation increases when the radius or azimuth increases. The latter is also confirmed by the linear correlations listed in Table 2.

The results indicate a significant weak positive correlation between the radius and the standard deviation, and a significant moderate positive correlation between the azimuth angle φ′ and the standard deviation. These results show that consistency of the controller drops with the distance (radius) and when the tracking objects are to the far left or far right (higher φ′) in the sensory space. Interestingly, no such dependency can be found when changing the inclination (θ) of the tracking objects.

**Figure 8.**
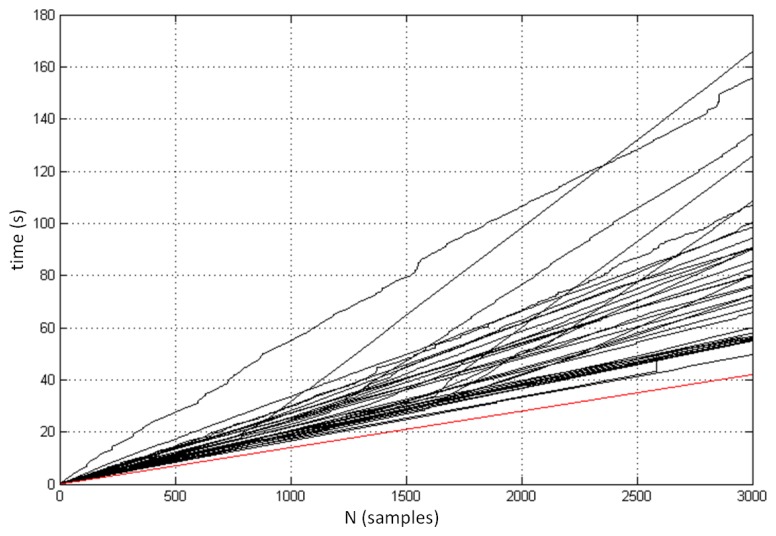
The progress of the measurements in the static scenario (the total time required to collect the initial 3,000 samples at different points in space).

**Figure 9.**
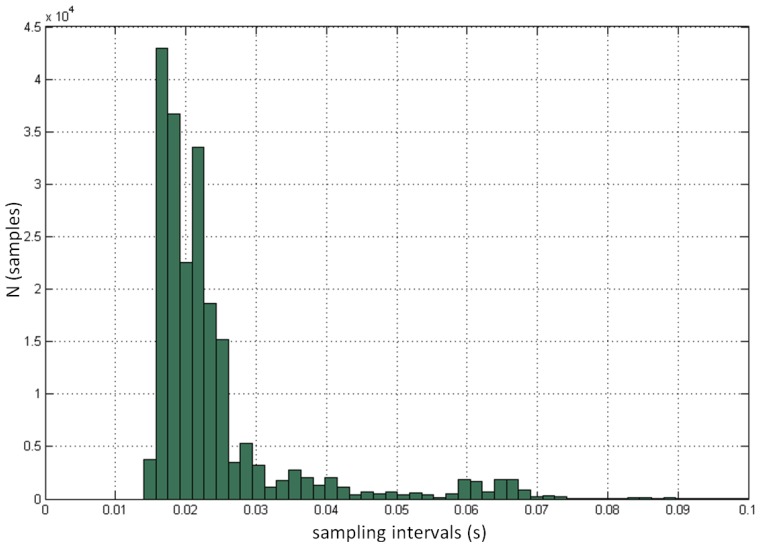
The distribution of the time intervals between two individual samples.

We also analyzed the sampling performance and sampling frequency of the controller. Each measurement of the controller was logged with the corresponding absolute timestamp, which enabled us to determine the exact time gap between two sequential samples and to calculate the corresponding sample frequency. Figure 8 demonstrates the progress of the measurements and the total time required to track the initial 3,000 samples for each of the 37 measured positions. It can be seen that sample frequency is very unstable and varies from measurement to measurement, and also within individual measurement. The minimal logged period between two samples was 14 ms (corresponds to sampling frequency 71.43 Hz). The red line at the bottom of the figure demonstrates the prediction of optimal sampling performance based on the highest measured sampling frequency. Figure 9 shows the distribution of the time intervals between two consecutive samples involving all 37 positions. The mean sampling frequency was 39.0 Hz. The standard deviation was 12.8 Hz.

### 4.2. Dynamic Measurements

**Figure 10.**
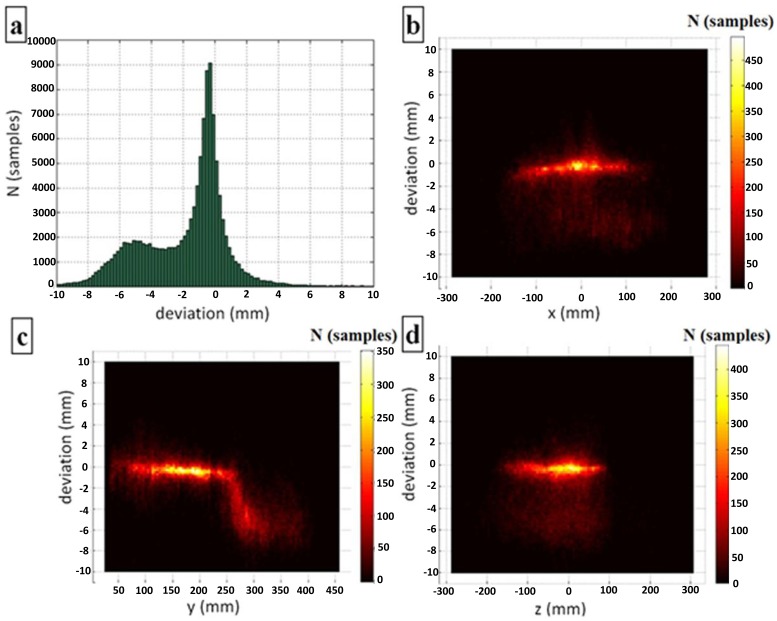
Distributions of deviation within dynamic measurements: (**a**) the overall distribution; and (**b**–**d**) the distributions on the individual axes.

A total of 119,360 measurements were taken within the dynamic measurement scenario in an attempt to cover the estimated useful sensory space of the controller, as described in the methodology section. Two markers with a constant inter-marker distance were used for tracking, and variations of that distance were used to analyze the controller's accuracy. Figure 10 demonstrates the distributions of the deviation of the distance. Figure 10a shows the overall distribution of samples for all the positions recorded by the controller. Figure 10b–d display the distributions of the deviation on the individual axes. In these cases, the brightness of the color indicates the density of the samples (higher brightness represents higher sample density).

The most interesting phenomenon, which can be noted in Figure 10a, is the non-Gaussian deviation distribution, which was not expected. In addition the global peak at a deviation of approximately 0 mm, another local peak is evident at a deviation of approximately −5 mm. Further analysis shows (Figure 10b–d) that this phenomenon originates in the measurements taken at *y* > 250 mm over the entire covered area of the *x*-*z* plane (−250 mm < *x*, *z* < 250 mm).

The analysis of the spatial dependency of the measured distance deviation was based on computing the correlations between the spatial dimensions and the distance (Table 3). The results reveal statistically significant moderate negative linear correlations between the distance deviation and the height above the controller (*y*) and the distance from the coordinate origin (*r*). The distance deviation is not correlated with the other spatial dimensions.

**Figure 11.**
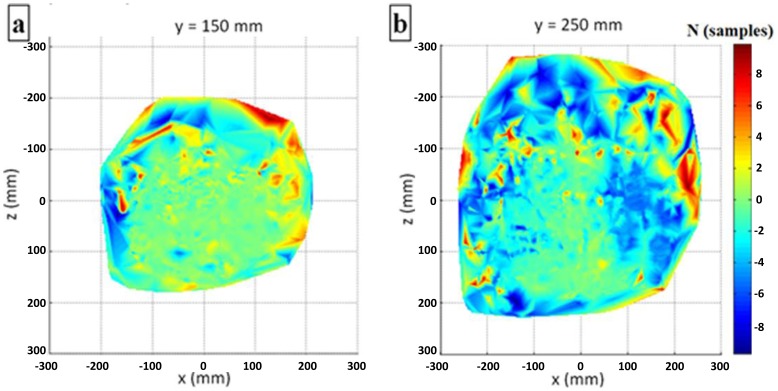
Distance deviation distributions in *x*-*z* plane at (**a**) *y* = 150 mm; and (**b**) *y* = 250 mm.

The additional volumetric analysis reveals the local distribution of the distance deviation on different planes. Figure 11 displays the deviation distribution on the *x*-*z* plane (different heights above the controller) at *y* = 150 mm (Figure 11a) and *y* = 250 mm (Figure 11b). The formerly presented “local peak anomaly” can be observed in Figure 11b, where the distance deviation tends towards lower values (blue color).

**Figure 12.**
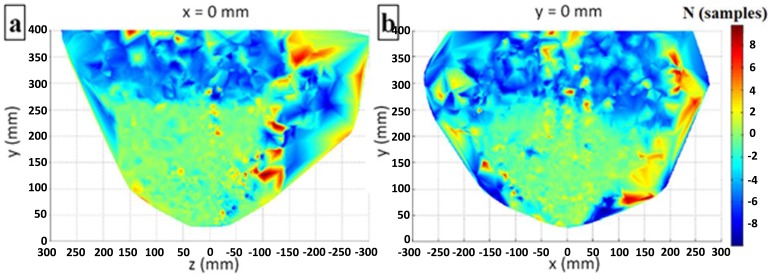
Distance deviation distributions at (**a**) *x* = 0; and (**b**) *y* = 0.

Figure 12 displays the deviation on the *x* = 0 (side view) and *z* = 0 (front view) planes. The figure reveals the highest deviation at the edges of the useful sensory space and at heights above *y* = 250 mm.

**Figure 13.**
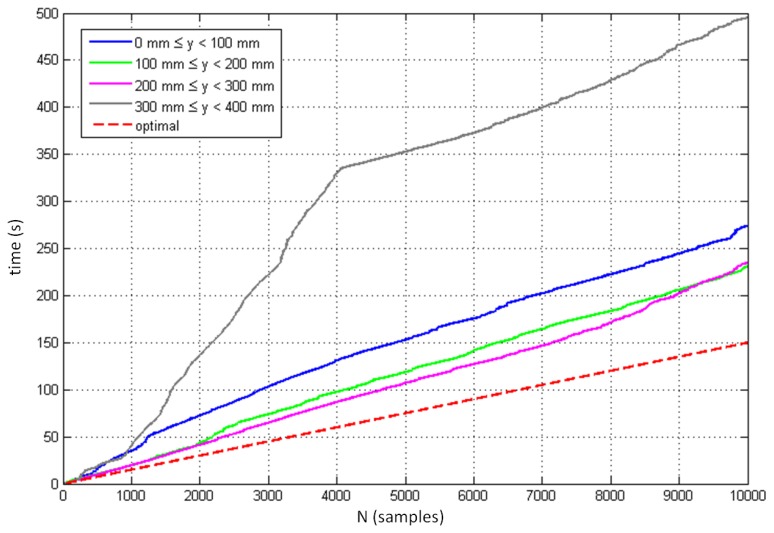
The progress of the measurements in the dynamic scenario (the total time required to collect the initial 10,000 samples in different height regions).

Figure 13 displays the controller's sampling performance when tracking moving objects in four different layers above the controller. The red broken line indicates the optimal sampling performance defined with a constant sampling period of 15 ms, which corresponds to the minimum time interval between two consecutive samples logged in the dynamic measurements. The figure indicates the best sampling performance between the heights of *y* = 100 mm and *y* = 300 mm, with significantly reduced efficiency above this height.

**Figure 14.**
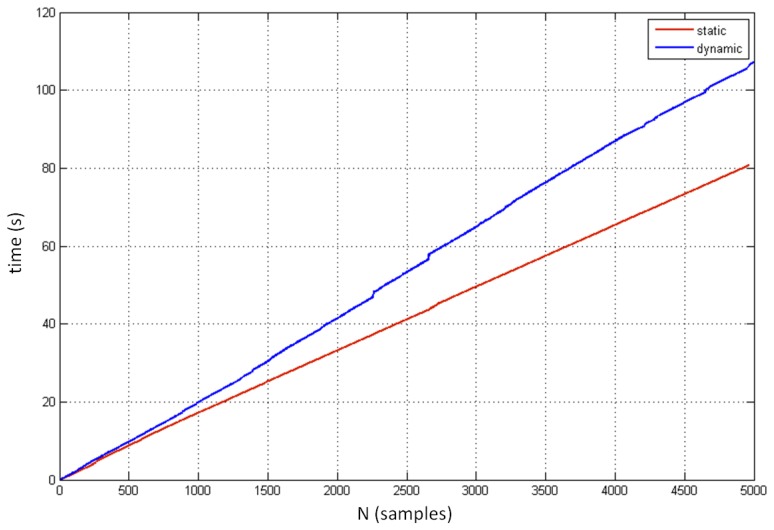
Comparison between the progress of static and dynamic measurements (total time required to collect the initial 5,000 samples).

Figure 14 compares the sampling performance for the static and dynamic conditions. The initial 5,000 samples of the best cases from both conditions were taken for this analysis. These results were expected, as the sampling performance in the static condition proved to be more robust and uniform than the performance in the dynamic condition.

## 5. Discussion and Conclusions

In this paper, we have described an extensive evaluation of the performance of the Leap Motion Controller with the aid of a professional fast and high-accuracy motion tracking system. The main goal of our research was to systematically analyze the controller's sensory space and to define the spatial dependency of its accuracy and reliability. We performed a set of static and dynamic measurements with different numbers and configurations of tracking objects.

In the static scenario, the standard deviation was shown to be less than 0.5 mm at all times, in the best cases less than 0.01 mm. In addition, the high accuracy (below 0.2 mm) reported in [15] combines with our results to evaluate the controller as a reliable and accurate system for tracking static points. Our analysis revealed an important spatial dependency of the controller's consistency and performance. The linear correlation revealed a significant increase in the standard deviation when moving away from the controller (radius) and when moving to the far left or right of the controller (φ′).

A sharp pen mounted on the robotic arm was used in [15], while we had to perform our measurements using a plastic arm with pointing finger. The algorithm of the controller seems to have been updated and requires a “hand-like” object to track static points. In many cases, we were unable to establish a stable environment, and the controller only tracked the static points for a few seconds and then stopped. The main criterion for choosing the final 37 spatial locations was, therefore, the establishment of a stable position for the tracking arm that enabled successful tracking and logging over a longer period of time. The majority of the successfully selected points were located behind the controller (*z* < 0), when the hand was located above the controller and therefore fully visible inside the sensory space. It was very difficult to set up the measurement when the hand was located in front of the controller and only the tracking finger remained in the sensory space.

The set of measurements in the dynamic scenario also revealed the inconsistent performance of the controller. Its accuracy was evaluated through the distortion of the distance between two moving points with a constant inter-point distance. In this case, the accuracy drops when the objects move away from the sensor. There is a significant drop in accuracy for the samples taken more than 250 mm above the controller. Due to this interesting and unexpected phenomenon, we repeated the measurement for this area and obtained the same results. It is impossible to speculate on the primary cause for this behavior, but perhaps the use of objects with different inter-object distances would reveal different results.

An important limitation of the controller's performance is its inconsistent sampling frequency. Its mean value of less than 40 Hz is relatively low and varies significantly under both static and dynamic conditions. The main drawback of the non-uniform sampling is the great difficulty to synchronize the controller with other real-time systems since it requires difficult post processing and re-sampling methods and operations.

Based on the insights gained from these experiments, the further study of the Leap Motion Controller may include research on the precision and reliability of tracking more complex hand/finger and tool movements as well as its suitability for applications strongly relying on gesture input modality.

The Leap Motion Controller undoubtedly represents a revolutionary input device for gesture-based human-computer interaction. In this study, we evaluated the controller as a possible replacement for a fast and high-precision optical motion capture system in a limited space and with a limited number of objects. Based on the current results and the overall experience, we conclude that the controller in its current state could not be used as a professional tracking system, primarily due to its rather limited sensory space and inconsistent sampling frequency.

## Figures and Tables

**Table 1.** Standard deviations of static positions.

**Standard deviation and position**		***x* Axis (std*_x_*)**	***y* Axis (std*_y_*)**	***z* Axis (std*_z_*)**	**Spatial position (std)**
**Minimal std**	std (mm)	**0.0081**	0.0093	0.015	0.013

	location (*x*, *y*, *z*) (cm)	(0, 30, 0)	(−10, 10, −5)	(0, 20, −5)	(0, 15, 0)

**Maximal std**	std (mm)	0.39	**0.49**	0.37	0.38

	location (*x*, *y*, *z*) (cm)	(−20, 20, 0)	(−20, 30, 0)	(−20, 30, 0)	(−20, 30, 0)

**Table 2.** Correlations between the dimensions of the spherical coordinate system and standard deviation.

**Correlation variables**	**Pearson coefficient**	***p*-value**
$\bar{p_{r}[\iota ]}$	0.338	0.044
$\bar{p_{\theta }[\iota ]}$	0.163	0.34
$\bar{p_{\varphi \prime }[\iota ]}$	0.433	0.051

**Table 3.** Correlations between spatial dimensions and the distance deviation.

**Correlation variables**	**Pearson coefficient**	***p*-value**
*x*[j], dist[j]	−0.0658	<0.000
*y*[j], dist[j]	**−0.612**	**<0.000**
*z*[j], dist[j]	0.00350	0.223
*r*[j], dist[j]	**−0.595**	**<0.000**
θ[j], dist [j]	0.192	<0.000
φ′ [j], dist [j]	−0.0792	<0.000
